# Evidence mapping based on systematic reviews of therapeutic interventions for gastrointestinal stromal tumors (GIST)

**DOI:** 10.1186/s12874-017-0402-9

**Published:** 2017-09-07

**Authors:** Mónica Ballesteros, Nadia Montero, Antonio López-Pousa, Gerard Urrútia, Ivan Solà, Gabriel Rada, Hector Pardo-Hernandez, Xavier Bonfill

**Affiliations:** 1 Iberoamerican Cochrane Centre, C/Sant Antoni Maria Claret,167, Pavelló 18, ground floor, 08025 Barcelona, Spain; 2 Centro de Investigación en Salud Pública y Epidemiología Clínica. Facultad de Ciencias de la Salud Eugenio Espejo, Universidad Tecnológica Equinoccial, Quito, Ecuador; 3 Oncología Médica y Unidad de Curas Paliativas, Hospital de la Santa Creu i Sant Pau, Barcelona, Spain; 4 Iberoamerican Cochrane Centre - Sant Pau Biomedical Research Institute, (IIB Sant Pau), CIBER Epidemiología y Salud Pública (CIBERESP), Universitat Autònoma de Barcelona, Barcelona, Spain; 5 Programa de Salud Basada en la Evidencia, Facultad de Medicina, Pontificia Universidad Católica de Chile, Santiago, Chile

**Keywords:** GIST, Gastrointestinal Stromal Tumours, Evidence mapping, Evidence synthesis, Global evidence mapping

## Abstract

**Background:**

Gastrointestinal Stromal Tumours (GISTs) are the most common mesenchymal tumours. Currently, different pharmacological and surgical options are used to treat localised and metastatic GISTs, although this research field is broad and the body of evidence is scattered and expanding. Our objectives are to identify, describe and organise the current available evidence for GIST through an evidence mapping approach.

**Methods:**

We followed the methodology of Global Evidence Mapping (GEM). We searched Pubmed, EMBASE, The Cochrane Library and Epistemonikos in order to identify systematic reviews (SRs) with or without meta-analyses published between 1990 and March 2016. Two authors assessed eligibility and extracted data. Methodological quality of the included systematic reviews was assessed using AMSTAR. We organised the results according to identified PICO questions and presented the evidence map in tables and a bubble plot.

**Results:**

A total of 17 SRs met eligibility criteria. These reviews included 66 individual studies, of which three quarters were either observational or uncontrolled clinical trials. Overall, the quality of the included SRs was moderate or high. In total, we extracted 14 PICO questions from them and the corresponding results mostly favoured the intervention arm.

**Conclusions:**

The most common type of study used to evaluate therapeutic interventions in GIST sarcomas has been non-experimental studies. However, the majority of the interventions are reported as beneficial or probably beneficial by the respective authors of SRs. The evidence mapping is a useful and reliable methodology to identify and present the existing evidence about therapeutic interventions.

**Electronic supplementary material:**

The online version of this article (doi:10.1186/s12874-017-0402-9) contains supplementary material, which is available to authorized users.

## Background

Sarcomas are rare malignant tumours of mesenchyme origin that occur in connective tissue. They can be split up into dozens of histological categories, which may develop at any age including childhood, can affect any anatomical localisation, and are of varying aggressiveness, even within the same histological subtype [1]. There are three main types of sarcoma corresponding to different clinicopathological entities which require a multidisciplinary approach: bone sarcomas, visceral (GIST being the most typical) and soft tissue sarcoma [2]. Gastrointestinal Stromal Tumours (GISTs) are the most common mesenchymal tumours [3, 4]. They constitute 1% to 3% of all malignant gastrointestinal tumours [5].

Classically, systematic reviews (SRs) summarise the results of available healthcare studies and provide a high amount of evidence on the effectiveness of healthcare interventions [6]. However, SRs frequently address very specific questions, preventing them from providing a comprehensive overview of a given topic [7, 8]. To overcome this barrier, new formats of review (e.g., scoping reviews, evidence map, rapid review, etc.) have been developed [9–11], allowing an understanding of the extent and distribution of evidence in a broad clinical area, highlighting both what is known and any gaps in evidence. [10].

In 2007, the Global Mapping Initiative (GEM) was established as a collaboration of clinical research and policy stakeholders to provide an overview of existing research about traumatic brain injury and spinal cord injury [12]. Evidence mapping is an emerging tool to systematically and comprehensively identify, organise and summarise the distribution of scientific evidence on any topic. It can be the first step to conduct systematic reviews or the framework to inform policy development [11–14].

The purpose of this evidence mapping project is to identify, describe and organise the current available evidence about therapeutic interventions on sarcomas. This approach aims to determine the clinical questions assessed in the scientific literature and the corresponding quality of the supporting evidence, as well as to give general information about their claimed effectiveness. This information shall facilitate detecting research gaps and help stakeholders in the decision-making process. For the sake of clarification, this publication focuses exclusively on GIST whereas the mapping of evidence on soft tissue sarcomas will be addressed in future publications.

## Methods

**Fig. 1:**
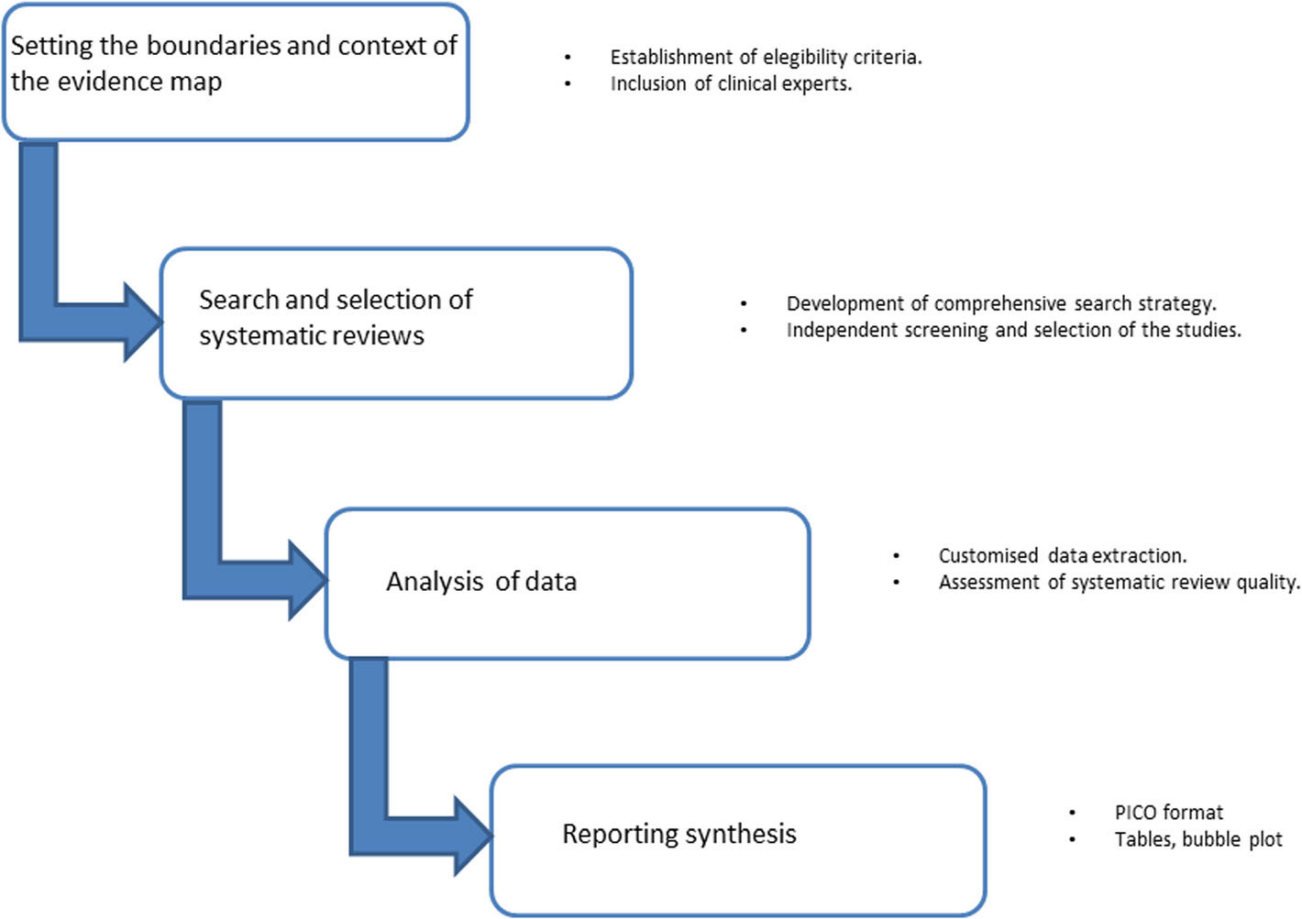
Tasks performed to map evidence in sarcomas

We conducted a mapping of evidence based on the methodology proposed by GEM [12]. In consequence, we did a comprehensive search strategy and assessed the quality of the included SRs. We have only included systematic reviews (with or without metanalysis) because they provide the most reliable empirical evidence in order to answer a specific research questions on therapeutic effects. We divided the process in four stages (Fig. 1: Tasks performed to map evidence in sarcomas).

### Setting the boundaries and context of the evidence map

In order to framework our mapping project, we consulted the 2013 World Health Organization (WHO) classification [15], and the related clinical guidelines, combined with the consultation to an oncologist with expertise in sarcomas. With this information we established the eligibility criteria for study inclusion. We selected SRs assessing therapeutic interventions in patients diagnosed with GIST, summarizing randomized controlled trials (RCTs) as well as phase I and II clinical trials, observational studies (including cohorts studies, case control studies, cases series), or case reports.

We used a broader definition of SR in order to obtain the largest possible number of documents. The systematic reviews that conducted a search in at least two databases were considered eligible. We included the most updated review if more than one version was identified. We excluded narrative reviews and systematic reviews that were focused on prognosis or cost-effectiveness. We also excluded studies on patients with Kaposi Sarcoma and/or Ewing’s tumours because of their unique biological characteristics and management [16].

The selection was done independently by two researchers.

### Searching and selection of systematic reviews

We conducted searches in PubMed, EMBASE, The Cochrane Library, and Epistemonikos from 1990 to March 2016; the former was updated in November 2016. The lower date boundary was chosen taking into account that biological mechanisms were discovered in 1998 and opened the use of biological agents as the key therapeutic approach of GIST that completely changed the management of the disease [17]. “However, we extended our retrospective search until 1990 for establishing a reasonable period of time to guarantee a higher sensitivity”.

We combined keywords and medical subject headings (Mesh terms) for all types of sarcoma according to the WHO 2013 classification [15]. We adapted the search strategy in accordance with the specific characteristics of each database. We did not limit searches by language. In addition, a clinical expert (AL) was consulted to help in identifying any other relevant reviews. Likewise, we reviewed all references in the relevant articles to identify potential additional reviews. Detailed search strategies are reported in Additional file 1.

We managed the search results with the reference manager software COVIDENCE [18]. After removing duplicates, two reviewers (MB, NM) independently screened all titles and abstracts to exclude irrelevant reviews. Full texts of potentially relevant reviews were obtained for a final decision. Disagreements were solved through discussion and consensus; if necessary, an additional reviewer (GU) was consulted. Reasons for exclusion are clearly stated.

### Data analysis

We built a data extraction form to register the main characteristics and quality of included systematic reviews. We tested a pre-defined data extraction form to ensure consistency among reviewers in a pilot study with 20% of eligible SRs. Two authors extracted data (MB, NM). Disagreements were solved by discussion with a third author (AL). We collected data at three levels:

- General characteristics from systematic reviews: authors, year of publication, type of systematic review (with or without meta-analysis), objective, search methods, design and number of included studies, type and number of patients included, and quality of the systematic review.

Two researchers (MB, NM) independently assessed the methodological quality of the included reviews with the AMSTAR tool. Disagreements were discussed until consensus was reached. We calculated AMSTAR scores by adding one point for each item rated as “yes” and no point for items rated as “no”, “cannot answer”, or “not applicable”, resulting in overall score ranging from 0 to 11. According to the total score, SRs were grouped in three categories: low quality (0 to 3 points), 4 to 7 points (moderate quality), and 8 to 11 points (high quality) [19].

**Fig. 2:**
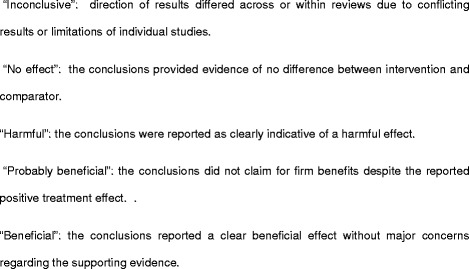
Classification of the conclusions according to results reported by authors

b) Clinical questions assessed in the systematic reviews: we converted the main aim reported in the included systematic review and their eligibility criteria into clinical questions framed in a PICO format (specifying the four key components: population, intervention, comparison and outcomes). The obtained PICOs were classified in five therapeutic scenarios with the help of a clinical expert (AL). We then extracted details about the population characteristics (e.g. adult population or children, type of sarcoma, localisation of tumours), the intervention and comparator (e.g. type of intervention and comparison broadly categorised as chemotherapy, surgery, radiotherapy and others, intention and temporality of the intervention, and comparison, drugs used in chemotherapy), and outcomes. For descriptive purposes, we also categorised the conclusions reported by the authors of the included studies, into five categories: “inconclusive”, “no effect”, “harmful”, “probably beneficial” and “beneficial” (see Fig. 2 to see the criteria followed for this categorization). Two authors completed this assessment independently (MB, NM); disagreements were solved by discussion until consensus was reached. In any case, this judgement represents a formal assessment about the evidence of interventions benefits and harms.
c) Characteristics of other research questions addressed in the systematic reviews, here named secondary PICOs: we defined secondary research questions as those for which all the elements of the PICO question were provided but the conclusions about the direction of the effect were described marginally in the article. We extracted the same information described above for the main research question.

### Synthesising findings

We adapted every clinical question addressed in each included review into a PICO format, which specifies the types of population (participants), types of interventions (and comparisons), and the types of outcomes of interest [6]. We classified PICO questions according to the disease stage (localised or unresectable and/or metastatic GIST) and summarised the findings for each included review using: a) tables describing the characteristics of the included systematic reviews, and another one with the characteristics of all PICOs identified (main and secondary), and b) graphic display of the mapping based on bubble plots. Each bubble in the chart represents one included systematic review. This chart displays information in three dimensions: (i) the rating of authors conclusions in the x-axis (“beneficial”, “probably beneficial”, “harmful”, “no effect”, and “inconclusive”) (which are further described in the data extraction section); (ii) the AMSTAR assessment in the y-axis, and (iii) bubble size according to the number of individual studies included in the SR. Each bubble is also a pie chart that shows the proportion of randomised controlled trials included in the SR through a black bold line.

## Results

**Fig. 3:**
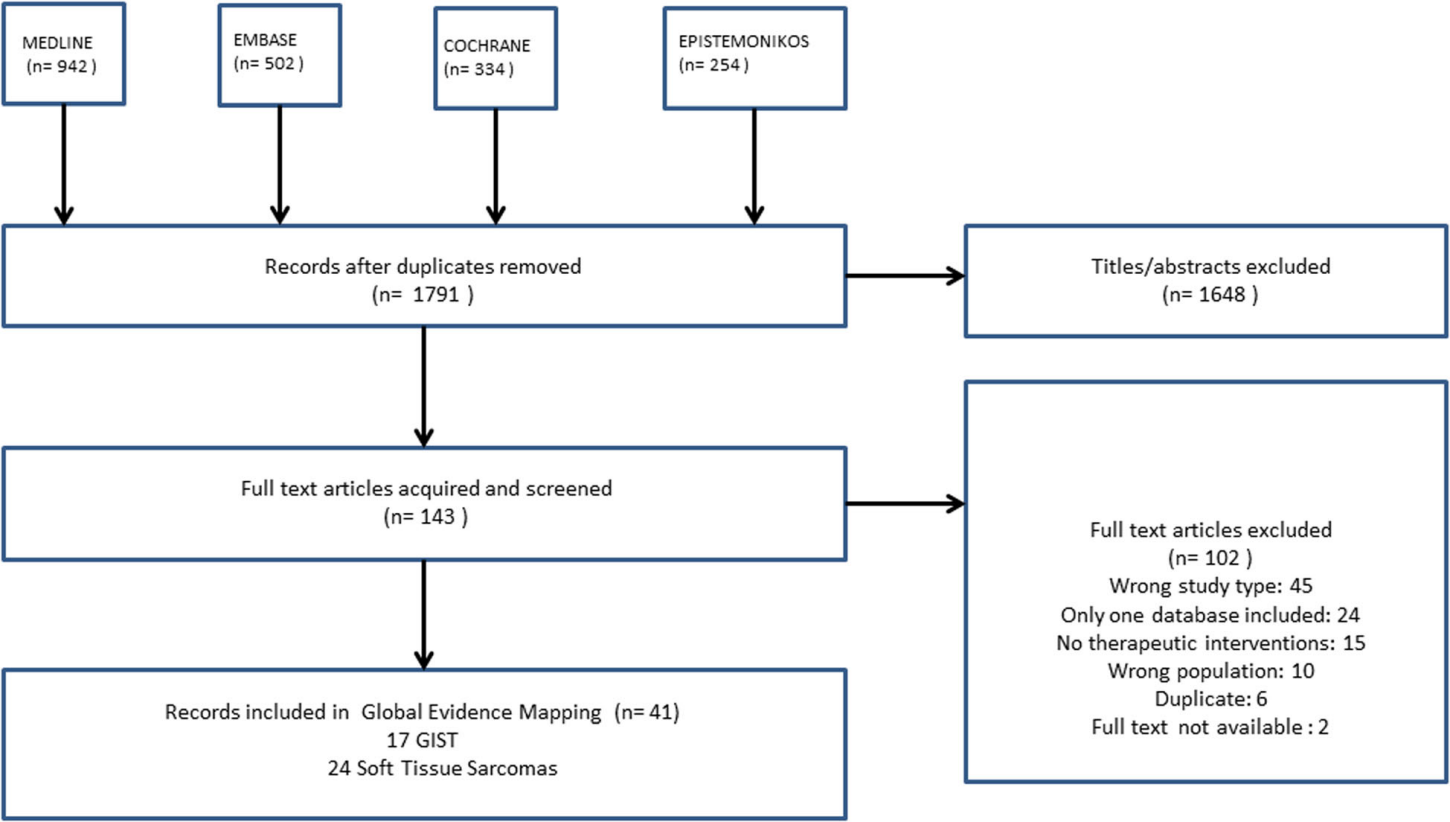
Flow chart outlining the study selection process

We obtained 1791 records from the search after removal of duplicates. Following screening of titles and abstracts, 143 articles were obtained in full text for a final decision. A total of 41 reviews fulfilled the inclusion criteria for the final analysis, of which 17 SRs are focused [17, 20–36] on GIST, which developed search strategies until 2014. A flow chart showing the selection of eligible reviews is presented in Fig. 3: Flow chart outlining the study selection process. The list of the 102 reviews excluded on the Evidence Mapping along with exclusion rationale is available in Additional file 2.

### Characteristics and quality of systematic reviews

Among the 17 included SRs, 10 included a meta-analysis. All SRs were published between 2005 and 2015 including studies conducted between 2001 and 2014. Only two SRs reported a detailed search strategy that allowed replication [23, 30]. A total of 66 individual studies were included in the SR after considering overlapping or duplication of studies, of which 43 were observational studies, 15 were randomised controlled trials, and 8 were phase II clinical trials.

**Fig. 4:**
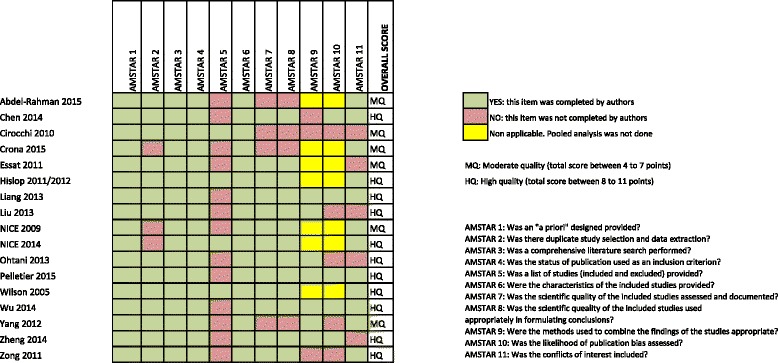
Quality of the included SRs

Seven systematic reviews did not include any controlled clinical trials [21, 23, 27, 30–32, 35], and among them one did not include any study [23]. The 10 remaining systematic reviews included at least one clinical trial. The number of patients included in the systematic reviews ranged from 233 to 2018 and all were adults. Twelve SRs were conducted to assess chemotherapy interventions [17, 20, 22–26, 28, 29, 32–34, 36] and five evaluated surgical interventions [21, 27, 30, 31, 35]. Only 3 of 12 SRs assessed chemotherapy with a curative intent [17, 24, 29], whereas in the remaining 9 SRs the chemotherapy had a palliative intent [20, 22, 23, 25, 26, 28, 32–34, 36]. All SRs on surgery stated a curative intent. All SRs assessed the clinical end-point and five reported surgical intermediate outcomes, such as, blood loss, earlier time to flatus, oral diet, etc. [21, 27, 30, 31, 35]. All SRs, except for two, reported overall survival [27, 35]; progression-free survival, response rate and local or distant recurrence rate were reported in seven reviews [17, 22, 25, 26, 28, 33, 34, 36]; and quality of life was assessed only in three SRs [22, 28, 32]. Two reviews reported data on adverse events [23, 33]. Overall, quality of the included SRs was moderate to high according to AMSTAR scores (Fig. 4: Quality of the included SRs). The most frequent drawbacks were: the failure to report the included and excluded studies [17, 20–22, 24, 27–31, 33–36], to declare possible conflicts of interest [17, 23, 24, 30, 35], to evaluate the likelihood of publication bias [17, 23, 30, 34, 36], and to assess bias of individual studies for using it appropriately in drawing conclusions [20, 22, 23, 34]. The characteristics of the included SR are summarised in Table 1.

**Table 1:** Summary of systematic reviews included in the Evidence Mapping

ID	Study design	Search methods and years	Research question or aim	Number of studies included	Type of study						Population/Type of GIST	Patients (N)	AMSTAR Score
					RCT	NRT	NCS	Cohort	CC	SC			
Abdel-Rahman 2015	SR	Standard search^a^; through July 2014.	To assess the available evidence for systemic therapy options for patients with advanced gastrointestinal stromal tumours beyond first-line imatinib.	26	6	0	15	5	0	0	Adults/Advanced GIST.	935	6
Chen 2014	SRM	Standard search; through June 2014.	To compare short-term and long-term results of patients undergoing laparoscopy surgery.	22	0	0	0	22	0	0	Adults/Localised GIST.	1166	9
Crona 2013	SR	Standard search; through August 2013.	To review the preclinical and clinical literature that led to the approval of regorafenib and to examine its place in therapy for the treatment of mCRC and GIST.	2	1	0	1	0	0	0	Adults/Advanced, unresectable and/or metastatic GIST.	233	5
Cirocchi 2010	SRM	Followed Cochrane Collaboration metodology; through June 2009.	To compare surgery alone with the use of imatinib as preoperative supplement for unresectable and/or metastatic GIST.	0	0	0	0	0	0	0	Adults/Unresectable and/or metastatic GIST.	901^a^	6
Essat 2011	SR	Standard search; through August 2009.	To evaluate the efficacy and safety of imatinib for the adjuvant treatment of adult patients with localised KIT (CD117)-positive GIST after complete surgical resection relative to current standard treatment (surgery without adjuvant therapy).	16	1	0	3	3	0	9	Adults/Localised GIST.	730	7
Hislop 2011/2012	SR	Standard search; through October 2010.	To determine the relative benefit (in patients who have acquired resistance to the 400 mg/day imatinib dose) of dose escalation (to either 600 mg/day or 800 mg/day imatinib), sunitinib or best supportive care.	5	3	1	0	1	0	0	Adults/ Unresectable and/or metastatic GIST.	669	9
Liang 2013	SRM	Standard search; through June 2012.	To compare surgical and oncologic outcomes of patients with GISTs undergoing laparoscopic resection surgery and open resection surgery.	17	0	0	0	17	0	0	Adults/Localised GIST.	776	10
Liu 2013	SRM	Standard search; through June 2012.	The current meta-analysis aimed to determine the efficacy and safety of different doses of imatinib in patients with GISTs and to identify the optimized dose, and to weigh the clinical benefit against the associated toxicity.	5	5	0	0	0	0	0	Adults/Unresectable and/or metastatic GIST.	2008	8
NICE 2009	SR	Standard search; through September 2008.	To consider the use of sunitinib malate for the treatment of people with unresectable and/or metastatic gastrointestinal stromal tumours (GIST) after failure of imatinib due to resistance or intolerance.	2	1	0	0	1	0	0	Adults/Unresectable and/or metastatic GIST.	1438	7
NICE 2014	SR	Standard search; through April 2014, updated on February 2014.	To consider the use of imatinib for the adjuvant treatment of gastrointestinal stromal tumours.	16	4	0	0	12	0	0	Adults/Unresectable and/or metastatic GIST.	2018	8
Ohtani 2013	SRM	Followed PRISMA recommendations; through June 2013.	To evaluate and compare the short- and long-term outcomes of laparoscopic and conventional open surgery for GIST.	12	0	0	0	12	0	0	Adults/Localized GIST.	644	8
Pelletier 2015	SRM	Standard search; through July 2012.	To compare the laparoscopic and open surgical resection of gastric GISTs to assess the effectiveness and safety of this minimally invasive technique.	7	0	0	0	6	1	0	Adults/Localized GIST.	330	10
Wilson 2005	SR	Standard search; through May 2003.	To assess the clinical and cost-effectiveness of imatinib in the treatment of unresectable and/or metastatic GISTs, relative to current standard treatments.	15	0	0	6	0	0	9	Adults/Unresectable and/or metastatic GIST.	1887	9
Wu 2014	SRM	Standard search; through February 2014.	To evaluate the efficacy of second-generation TKIs with regard to progression-free survival and overall survival in patients with advanced GIST.	3	3	0	0	0	0	0	Adults/Advanced GIST.	808	10
Yang 2012	SRM	Standard search; through February 2012.	To evaluate the efficacy and safety of two doses of imatinib for patients with GISTs.	5	5	0	0	0	0	0	Adults/Unresectable and/or metastatic GIST.	1861	7
Zheng 2014	SRM	Standard search; through May 2012.	To assess existing evidence about the efficacy and safety of laparoscopic resection versus that of open resection for gastric GISTs.	11	0	0	0	11	0	0	Adults/Localized GIST.	495	9
Zong 2011	SRM	Standard search; through July 2010.	To evaluate the response to imatinib at different dose levels to identify the better choice for the treatment of patients with GIST.	3	3	0	0	0	0	0	Adults/Unresectable and/or metastatic GIST.	1787	9

^a^No controlled or randomised clinical trials comparing surgery alone and use of imatinib as preoperative supplement were available when the SR was conducted. The authors performed a subgroup analysis in the patients receiving preoperative treatment with imatinib mesylate (*N* = 901)

● Standard search: Strategies documented in detail enough to be replicated

RCT: Randomized controlled trial; NRT: Non-randomized trial; NCS: Non-controlled trial; CC: Case-control study; CS: case series

### PICO questions included in systematic reviews

We extracted 14 PICO questions from related to GIST SRs. The key characteristics of the PICOs are presented in Table 2. Depending on the specific type of GIST, the PICOs were grouped in the following five clinical scenarios, which include the entire clinical spectrum of the disease (from non-metastatic to metastatic cancer):

1. Patients with localised GIST: Five systematic reviews [21, 27, 30, 31, 35] with a total of 28 observational studies and no RCTs. All compared laparoscopic resection versus open resection in GIST adult patients. In general, the analysed outcomes were related to surgery results (blood loss, time to flatus, operative time, time to oral intake, length of hospital stay, complication rate) and oncology outcomes (overall survival, disease-specific survival). The overall conclusion from SRs was in favour of laparoscopic resection and the effects were categorised as “beneficial” [27, 30], and “probably beneficial” [21, 31, 35] due to concerns about the lack of clinical trials.
2. Patients with localised KIT (CD117)-positive GIST after complete surgical resection: two SRs [24, 29] which addressed the question of whether imatinib should be given as adjuvant treatment versus surgery alone without imatinib. In accordance with the risk of recurrence, one SR assessed adjuvant imatinib for overall population (all risk categories divided in three subgroups: high, intermediate, and low) [24] and the other focused on high-risk patients [29]. These two SRs based their conclusions on two controlled trials, six uncontrolled trials and nine observational studies. Overall, the results from the included reviews favoured adjuvant imatinib for patients at intermediate and high risk of recurrence, and the conclusions could be categorised as “beneficial” [24] and “probably beneficial” [29], respectively. For patients at low risk of recurrence, the conclusion of one systematic review [24] was rated as “no effect” based on the subgroup analysis of one controlled trial. One of these SR [29] also evaluated the duration of adjuvant imatinib in this population, and qualified the use of adjuvant imatinib for ≥3 years as “probably beneficial” based on one controlled trial and one observational study.
3. Patients with unresectable and/or metastatic GIST: five SRs evaluated different comparisons [17, 23, 32, 34, 36]. One SR assessed imatinib versus other standard treatments (these included interventions for symptom relief, best supportive care and placebo) and did not find controlled trials that directly evaluated this comparison [32]. Based on indirect comparison with six uncontrolled trials and one observational study, the use of imatinib in this population was classified as “probably beneficial”. One SR assessed the use of preoperative imatinib in the same population and compared it with surgery alone, but no eligible study addressing this issue was found [23]. Three SR [17, 34, 36] with five RCTs in this category assessed if high versus standard doses of imatinib should be used. Overall, the high imatinib doses were considered as “harmful” due to a misbalance between benefits and harms.
4. Patients with unresectable and/or metastatic GIST after failure of imatinib due to resistance or intolerance: four SRs addressing three different comparisons [20, 25, 26, 28, 33]. Two SRs assessed Sunitinib plus best supportive care versus imatinib at escalated doses [20, 25, 26]. No studies directly assessing this comparison were found. These two SRs presented inconsistent conclusions based on indirect comparisons from three trials and four observational studies: “beneficial” in one SR [20] and “no effect” in the other [25, 26]. One was Sunitinib plus best supportive care (as defined by the respective authors) versus best supportive care or placebo. This comparison was assessed in three SRs including one controlled trial and one observational study [20, 28, 33]. Sunitinib plus best supportive care was categorised as “beneficial” [28] and “probably beneficial” [20, 33] for this population due to limitations in the included studies. The third comparison, masitinib versus sunitib was assessed in one systematic review based on one controlled trial, which concluded that masitinib is “probably beneficial” [20].
5. Patients with unresectable and/or metastatic GIST after failure of imatinib and sunitinib due to resistance or intolerance: three SRs comprising three different comparisons [20, 22, 33]: a) Resumption of imatinib versus placebo was rated as “probably beneficial” in one systematic review including one controlled trial [20]; b) regorafenib plus best supportive care versus best supportive care or placebo was rated as “probably beneficial” in three SRs including one controlled trial and one uncontrolled trial [20, 22, 33]; and c) nilotinib versus placebo was rated as “no effect” and “inconclusive” by two SRs including the same controlled trial [20, 33].

**Table 2:** PICOs included on systematic reviews

Population	Intervention	Comparison	Outcomes	Systematic Reviews included	Individual studies included in the systematic review			Conclusion
					Controlled trials	Uncontrolled trials	Observational studies	
Patients with localized GIST	Laparoscopic resection	Open resection	Surgery outcomes: blood loss, time to flatus, operative time, time to oral intake, length of hospital stay, complication rate. Oncology outcomes: overall survival, disease-specific survival, local and metastatic recurrence.	Chen 2014, Liang 2013, Othani 2013, Pelletier 2015, Zheng 2014			Catena 2008, Dai 2011, Goh 2010, Ishikawa 2006, Karakousis 2011, Kasetsermwiriya 2014, Kim 2012, Lee 2011, Lee 2013, Lin 2014, Matthews 2002, Melstrom 2012, Mochizuki 2006, Nishimura 2007, Pitsinis 2007, Pucci 2012, Shimizu 2002, Silberhumer 2009, Shu 2013, Takahashi 2014, De Vogelaere 2013, Wan 2012, Wang 2010, Wu 2010, Xu 2009, Zhao 2011, Zhou 2009, Zong 2011	Beneficial Probably beneficial
Patients with localized KIT (CD117)-positive GIST after complete surgical resection High risk of relapse after surgery Intermediate risk of relapse after surgery Low risk of relapse after surgery	Imatinib adjuvant	Surgery alone	overall survival, recurrence-free survival, recurrence rates, adverse effects	Essat 2011	ACOSOG Z99001 (DeMatteo 2009)	ACOSOG Z9000 (DeMatteo 2005), Zhan2006.	Bumming 2003, Kang 2009, Li 2009, Nilsson 2007	Beneficial
	Imatinib adjuvant	Surgery alone	overall survival, recurrence Seddon2008.free survival, adverse events	Essat 2011, NICE 2014	ACOSOG Z99001 (DeMatteo 2009, Corless 2010), EORTC 62024 (Casali 2013)	ACOSOG Z9000 (DeMatteo 2005), Nisihda 2009, Yalcin 2012, Zhan 2006	Bumming 2003, Jiang 2011, Kanda 2013, Kang 2013, Li 2009, Li 2011, Nilsson 2007	Beneficial Probably beneficial
	Imatinib adjuvant for ≥3 years	Imatinib adjuvant <3 years	Overall Survival, local recurrence	NICE 2014	SSGXVIII/AIO (Joensuu 2012, Heintz 2011),		Conley 2012,	Probably beneficial
	Imatinib adjuvant	Surgery alone	Recurrence- free survival	Essat 2011	ACOSOG Z99001^b^ (DeMatteo 2009)			Beneficial
	Imatinib adjuvant	Surgery alone	Recurrence- free survival	Essat 2011	ACOSOG Z99001 (DeMatteo 2009)			No effect
Patients with unresectable and/or metastatic GIST	Imatinib	Current standard treatments	Overall survival, response rate, quality of life, adverse events	Wilson 2005		Benjamin 2003^a^ (interinm results of Blanke Rankin 2008), Demetri 2002^a^, Judson 2003, Ryu 2003, Van Oosterom2001, Verweij 2004	Jankilevich 2003	Probably beneficial
	Preoperative imatinib + surgery	Surgery alone	Overall survival, disease-free survival	Cirocchi 2010	No studies			Inconclusive
	Imatinib high dose Imatinib 800 mg /day Imatinib 600 mg/day	Imatinib standard dose Imatinib 400 mg /day Imatinib 400 mg /day	Overall survival, progression free survival, response rate, dose reduction, adverse events Overall survival, progression free survival, adverse events Overall survival, response rate, adverse events	Liu 2013, Yang 2012, Zong 2011 Liu 2013 Liu 2013	Blanke Demetri 2008,Blanke Rankin 2008, Dagher 2002, Demetri 2002, Nishida 2008, Verweij 2004 Blanke Rankin 2008, .Verweij 2004 Dagher 2002, Demetri 2002, Nishida2008			Harmful Harmful Harmful
Patients with unresectable and/or metastatic GIST after failure of imatinib due to resistance or intolerance.	Sunitinib + BSC	Imatinib at escalated doses	Overall survival, progression free survival, response rate and adverse effects.	Abdel- Rahman2015, Hislop 2011/2012		EORTC-ISG-AGITG (Zalcberg 2005, Debiec Rychter 2006), Blanke Rankin 2008^a^, B2222^a^ (Blanke Demetri 2008, Demetri 2006)	Hsu 2014, Vincenzi 2014, Park2009, Seddon2008.	Probably beneficial No effect
	Sunitinib + BSC	BSC/ placebo	Overall survival, progression free survival, response rate, quality of life, adverse events	NICE 2009, Abdel-Rahman 2015, Wu 2014	Demetri 2006 (interim of Demetri 2012), Demetri 2012		Reichardt 2008	Beneficial Probably beneficial
	Masinitib	Sunitinib	Overall survival	Abdel –Rahman 2015	Adenis 2014			Probably beneficial
Patients with unresectable and/or metastatic GIST after failure of imatinib and sunitinib due to resistence or intolerance	Resumption of previous imatinib 400 mg	Placebo	Overall survival, progression free survival, response rate, adverse effects.	Abdel Rahman 2015	Kang 2013			Probably beneficial
	Regorafenib + BSC	BSC/ placebo	Overall survival, progression free survival, and adverse effects.	Abdel-Rahman 2015, Crona 2013, Wu 2014)	Demetri 2013	George 2012		Probably beneficial
	Nilotinib	Placebo	Overall survival, progression free survival, response rate, adverse effects.	Abdel-Rahman 2015, Wu 2014	Reichardt 2012			No effect Inconclusive

^a^Controlled trials, however for this PICO were used to formulate indirect comparisons

^b^Although the trial was not designed to assess patient subgroups, authors presented results for three different groups: high, intermediate and low risk

## Discussion

Although no standard definition of evidence mapping has emerged [11], these reviews share some common characteristics: (a) they are appropriate for addressing broad topics that are often too expansive for an individual systematic review; (b) they involve experts in the area of study to set the inclusion and exclusion criteria; (c) they are based on a systematic search; and (d) they include user-friendly summaries of results.

Following these criteria, this evidence mapping has identified, described and organised the current available evidence for GIST, as part of a broader project aimed to map the existing evidence for the treatment of soft-tissue sarcomas. This mapping was based on 17 published systematic reviews including 66 individual studies conducted between 2001 and 2014. Regardless of the type of evaluated intervention, three quarters of the included studies in the SR were non-experimental (observational studies or uncontrolled clinical trials). This is a phenomenon with important clinical and ethical implications since experimental studies are the best design to evaluate the efficacy of new therapeutic options. For instance, it is noteworthy that some clinical guidelines or systematic reviews [3, 23, 32, 37–39] are already considering surgery resection as the current standard of care for localised GIST. However, according to the SR included in this evidence mapping, none of the studies used to support that recommendation about surgery were randomised controlled trials; the total number of included patients was less than 1000; and the results consisted of intermediate surgery-related end-points rather than patient-centred outcomes. Another example is the use of imatinib, a new biologic agent, in patients with unresectable and/or metastatic GIST, evaluated in one SR (37) that only included uncontrolled trials and one observational study.

The majority of the interventions reported as “beneficial” were palliative, probably due to a high proportion of patients experiencing relapse or a metastatic process. Another interesting finding was that only three studies assessed the quality of life as an outcome and none of them conducted an economic evaluation. Quality of life measures are very important in cancer care because they can provide information about the impact of diseases and their treatment on the well-being of patients, and complements efficacy and safety data [40]. Likewise, the economic evaluation contributes to allocating resources within society as efficiently as possible [41].

**Fig. 5:**
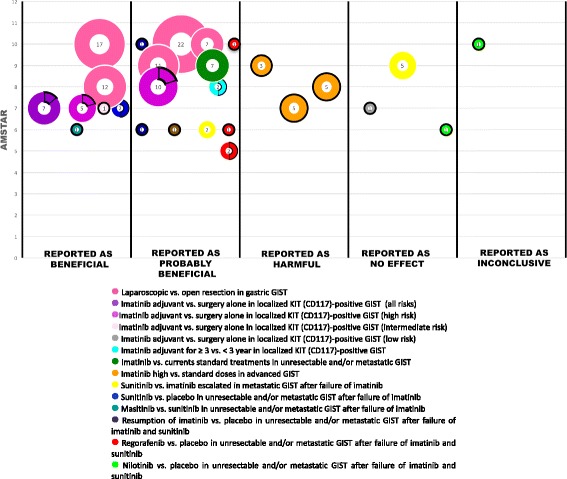
Mapping of evidence of GIST

The majority of the interventions were reported by authors as “beneficial” or “probably beneficial”. Only in one comparison between biologic agents (sunitinib versus imatinib escalated doses in unresectable and/or metastatic GIST after failure of imatinib) were the results controversial. As shown in the bubble plot, (Fig. 5) one SR [20] concluded that sunitinib is “probably beneficial” over imatinib at escalated doses, whereas another one [25, 26] considered that sunitinib has no effect on this type of patients. This discrepancy may be due to the fact that Hislop SR [25, 26] was based on indirect comparisons from small phase II non-randomised studies, whereas Abdel-Rahman SR [20] evaluated two retrospective observational comparative studies with direct comparisons. Currently, sunitinib is usually recommended after failure of escalated doses of imatinib for these type of patients [5, 38, 42, 43].

Overall, the quality of the SRs according to AMSTAR was moderate to high. However, the following domains have yet to be improved: reporting the excluded studies (only 4 studies out of 17 did it), the conflicts of interest, and the assessment of the likelihood of publication bias (only 12 did it). Similarly, 4 SRs did not report the quality of the included studies nor use it appropriately in formulating conclusions, one of them being (31) the review with more studies, the second one with more included patients, and the most updated evidence about metastatic GIST after failure of imatinib and sunitinib due to resistance or intolerance. Although the evidence mapping does not usually include a quality assessment process [9], we consider that any typology of review (e.g. rapid review, scoping review, umbrella review) should evaluate this aspect in order to assess the reliability of the conclusions; particularly in this case, where most studies included in SRs on GIST sarcomas are non-experimental and have small sample sizes.

### Strengths

The Global Evidence Mapping Initiative (GEM) approach that we used is a rational, systematic and constantly improved methodology. A recent systematic review [11] showed that among the 16 documents that met the common characteristics of evidence mapping, seven referenced the GEM.

Some authors consider using highly specific search strategies for evidence mapping [44, 45]. However, for the purposes of our project, we preferred to make a sensitive and adapted search strategy, taking into account the fourth edition of the World Health Organization Classification of Tumours of Soft Tissue and Bone [15] and the clinical background and expertise of one of the members of the research group, which revealed to be of great value for clarifying content doubts. Likewise, we used a broad definition of systematic review in order to obtain the largest number of documents. Thus, we consider that our search strategy is comprehensive as well reproducible; hence, it is unlikely that any relevant systematic review on sarcomas has been missed. We used the PICO format to organise and classify the information obtained from SRs, which was very useful in establishing thematic areas in this broad field. We also organised the results in graphical formats and corroborated them with other related clinical documents (e.g. clinical guidelines and consensus). Following the recommendations of GEM, we used two data extraction methods [12]: general (for characteristics of included systematic reviews) and specific (for main and secondary PICOs).

We added two uncommon components in evidence mapping. Firstly, we rated the interventions included in the systematic reviews as “beneficial”, “probably beneficial”, “harmful”, “no effect” or “inconclusive” according to the authors’ conclusions, irrespective of the reported outcomes. It is important to highlight that we did not evaluate the quality of the evidence of the studies included in each SR, which makes this approach shorter than the one required by a SR and appropriate for its descriptive purposes although the provided information is less complete. Secondly, we assessed the quality of included systematic reviews with AMSTAR. This approach allows displaying the results on a bubble plot for each SR with respect to the other ones with the same comparison, providing a quick view of the existing evidence and their quality.

According to this experience, the most time-consuming phases were classifying the interventions and extracting the secondary PICOs. Although the time spent in an evidence mapping can vary depending on the topic, we recommend elaborating a protocol before starting the project and performing a pilot study as we did.

### Limitations

Some limitations were faced in this study. Firstly, our search for SRs was conducted in 2016 but their respective searches were done much earlier, being the most updated search until 2014. Therefore, we cannot guarantee a comprehensive identification of all primary studies about sarcomas that may have been published beyond this date. However, we believe that these limitations would not substantially change the main results of this evidence mapping. Secondly, as it is a characteristic of all evidence mapping methodologies, we did not assess the quality of the evidence supporting the conclusions, which would have required the use of some complementary criteria such as GRADE [46]. In order to provide some qualitative information about the validity of SR, we assessed them through AMSTAR, which is a validated tool [19]. However, a noteworthy drawback of our evidence mapping is that it merely organises and describes the available evidence as is reported by respective authors. This explains why many treatments are presented as beneficial even they are based on non-experimental studies.

Therefore, the main practical applications of evidence mapping are: to orientate further research projects; to stimulate the design of more focused RCT and other rigorous evaluative studies to fill the detected gaps in knowledge; to provide useful comprehensive information for establishing priorities when funding research in this field; to compare the obtained results with the recommendations from clinical guideline in order to identify and solve potential contradictions between them; to help future authors of SRs, rapid reviews and scoping reviews avoiding redundant efforts and improve efficiency; and, to explore innovative tools and friendly formats to disseminate the results to interested stakeholders.

## Conclusions

From a practical point of view, this evidence mapping shows a relatively high consistency of effects reported by the different SRs, except for two SRs (comparing sunitinib versus escalated imatinib in GIST metastatic patients after failure of imatinib) (Fig. 5). The quality of the included SRs based on the AMSTAR criteria is moderate to high, which gives some confidence about the validity of their results. The scarce number of clinical trials in this field is remarkable, and we consider that the most important clinical questions have been covered.

In conclusion, the most common type of study to evaluate therapeutic interventions in GIST sarcomas has been non-experimental studies (observational studies or uncontrolled clinical trials), frequently based on small samples sizes. The quality of the included SR was moderate to high. The evidence mapping is a useful and reliable methodology to identify and present the current available evidence about therapeutic interventions. Therefore, these results can be helpful to facilitate any review process that may be conducted and orientate research priorities.

## Additional files

Additional file 1: Search strategies. (DOCX 44 kb)

Additional file 2: SRs excluded. (DOCX 18 kb)

## Abbreviations

AMSTAR: Assessing the Methodological Quality of Systematic Reviews
GEM: Global Evidence Mapping Initiative
GIST: Gastrointestinal Stromal Tumours
PDGFR alpha: Platelet Derived Growth Factor Receptor Alpha
PICO: Population, Intervention, Comparison, Outcome
RCT: Randomized Controlled Trial
SRs: Systematic Reviews
WHO: World Health Organization
