# Supplementary material for: Evidence mapping based on systematic reviews of therapeutic interventions for gastrointestinal stromal tumors (GIST)
Source: BMC Med Res Methodol. 2017 Sep 7;17:135. doi: 10.1186/s12874-017-0402-9 (PMC5590134; doi:10.1186/s12874-017-0402-9)
Supplement: Supplementary file 1 — Search strategies. (DOCX 44 kb) [file 12874_2017_402_MOESM1_ESM.docx]

**Additional file 1**

Search Strategy: Pubmed

| Search | Add to builder | Query | Items found |
| --- | --- | --- | --- |
|  |  | Search ((((((((((((((((((((((((((((((((((((((((((((((((((((((((((((((sarcoma*[Title/Abstract]) OR sarcoma[mh:noexp]) OR liposarcoma*) OR "Liposarcoma"[Mesh]) OR "Fibrosarcoma"[Mesh]) OR fibrosarcoma*) OR dermatofibrosarcoma*[Title/Abstract]) OR myxofibrosarcoma*[Title/Abstract]) OR "Myxosarcoma"[Mesh]) OR myxosarcoma*[Title/Abstract]) OR haemangiopericytoma*[Title/Abstract]) OR hemangiopericytoma*[Title/Abstract]) OR "Hemangiopericytoma"[Mesh]) OR histiocytoma*[Title/Abstract]) OR hystiocytoma*[Title/Abstract]) OR "Histiocytoma, Malignant Fibrous"[Mesh]) OR MFH[Title/Abstract]) OR "Leiomyosarcoma"[Mesh]) OR leiomyosarcoma*[Title/Abstract]) OR glomangiosarcoma*[Title/Abstract]) OR "Myosarcoma"[Mesh]) OR rhabdomyosarcoma*[Title/Abstract]) OR "Hemangioendothelioma"[Mesh]) OR haemangioendothelioma*[Title/Abstract]) OR hemangioendothelioma*[Title/Abstract]) OR "Hemangiosarcoma"[Mesh]) OR hemangiosarcoma*[Title/Abstract]) OR haemangiosarcoma*[Title/Abstract]) OR angiosarcoma*[Title/Abstract]) OR "Lymphangiosarcoma"[Mesh]) OR lymphangiosarcoma*[Title/Abstract]) OR "Gastrointestinal Stromal Tumors"[Mesh]) OR "gastrointestinal stromal tumor"[Title/Abstract]) OR "gastrointestinal stromal tumors"[Title/Abstract]) OR GIST[Title/Abstract]) OR ectomesenchymoma*[Title/Abstract]) OR "malignant granular cell tumor"[Title/Abstract]) OR MPNST[Title/Abstract]) OR "Mesenchymoma"[Mesh]) OR mesenchymoma*[Title/Abstract]) OR "Sarcoma, Synovial"[Mesh]) OR synovial sarcoma*[Title/Abstract]) OR "sarcoma synovial"[Title/Abstract]) OR synovioma*[Title/Abstract]) OR ASPS[Title/Abstract]) OR "Sarcoma, Clear Cell"[Mesh]) OR "clear cell sarcoma"[Title/Abstract]) OR "clear cell sarcomas"[Title/Abstract]) OR "Desmoplastic Small Round Cell Tumor"[Mesh]) OR "Desmoplastic small round"[Title/Abstract]) OR "Rhabdoid Tumor"[Mesh]) OR "Rhabdoid Tumor"[Title/Abstract])) OR (((((((((((("tumor rhabdoid") OR PEComa[Title/Abstract]) OR "Perivascular Epithelioid Cell Neoplasms"[Mesh]) OR "perivascular epithelioid cell"[Title/Abstract]) OR uterine sarcoma*[Title/Abstract]) OR ovarian sarcoma*[Title/Abstract]) OR "sarcoma ovarian"[Title/Abstract]) OR "fibrohistiocytic tumor"[Title/Abstract]) OR "fibrohistiocytic tumour"[Title/Abstract]) OR myofibroblastic sarcoma*[Title/Abstract]) OR fibromyxoid sarcoma*[Title/Abstract]) OR epithelioid fibrosarcoma*[Title/Abstract])))) OR "endometrial sarcoma"[Title/Abstract]) OR "endometrial stromal"[Title/Abstract]) OR "sarcoma, endometrial stromal"[MeSH Terms])) AND systematic[sb])) NOT (((kaposi*[Title]) OR Ewing*[Title])) Filters: Publication date from 1990/01/01 to 2015/12/31 | [947](http://www.ncbi.nlm.nih.gov/pubmed/?cmd=HistorySearch&querykey=1) |
| [#139](http://www.ncbi.nlm.nih.gov/pubmed/advanced) | [Add](http://www.ncbi.nlm.nih.gov/pubmed/advanced) | Search (kaposi*[Title]) OR Ewing*[Title] | [11860](http://www.ncbi.nlm.nih.gov/pubmed/?cmd=HistorySearch&querykey=139) |
| [#138](http://www.ncbi.nlm.nih.gov/pubmed/advanced) | [Add](http://www.ncbi.nlm.nih.gov/pubmed/advanced) | Search Ewing*[Title] | [4085](http://www.ncbi.nlm.nih.gov/pubmed/?cmd=HistorySearch&querykey=138) |
| [#137](http://www.ncbi.nlm.nih.gov/pubmed/advanced) | [Add](http://www.ncbi.nlm.nih.gov/pubmed/advanced) | Search kaposi*[Title] | [7775](http://www.ncbi.nlm.nih.gov/pubmed/?cmd=HistorySearch&querykey=137) |
| [#135](http://www.ncbi.nlm.nih.gov/pubmed/advanced) | [Add](http://www.ncbi.nlm.nih.gov/pubmed/advanced) | Search systematic [sb] | [279315](http://www.ncbi.nlm.nih.gov/pubmed/?cmd=HistorySearch&querykey=135) |
| [#131](http://www.ncbi.nlm.nih.gov/pubmed/advanced) | [Add](http://www.ncbi.nlm.nih.gov/pubmed/advanced) | Search "sarcoma, endometrial stromal"[MeSH Terms] | [4128](http://www.ncbi.nlm.nih.gov/pubmed/?cmd=HistorySearch&querykey=131) |
| [#128](http://www.ncbi.nlm.nih.gov/pubmed/advanced) | [Add](http://www.ncbi.nlm.nih.gov/pubmed/advanced) | Search "endometrial stromal"[Title/Abstract]) | [27579](http://www.ncbi.nlm.nih.gov/pubmed/?cmd=HistorySearch&querykey=128) |
| [#125](http://www.ncbi.nlm.nih.gov/pubmed/advanced) | [Add](http://www.ncbi.nlm.nih.gov/pubmed/advanced) | Search "endometrial sarcoma"[Title/Abstract]) | [85460](http://www.ncbi.nlm.nih.gov/pubmed/?cmd=HistorySearch&querykey=125) |
| [#124](http://www.ncbi.nlm.nih.gov/pubmed/advanced) | [Add](http://www.ncbi.nlm.nih.gov/pubmed/advanced) | Search epithelioid fibrosarcoma*[Title/Abstract] | [81](http://www.ncbi.nlm.nih.gov/pubmed/?cmd=HistorySearch&querykey=124) |
| [#118](http://www.ncbi.nlm.nih.gov/pubmed/advanced) | [Add](http://www.ncbi.nlm.nih.gov/pubmed/advanced) | Search myofibroblastic sarcoma*[Title/Abstract] | [112](http://www.ncbi.nlm.nih.gov/pubmed/?cmd=HistorySearch&querykey=118) |
| [#115](http://www.ncbi.nlm.nih.gov/pubmed/advanced) | [Add](http://www.ncbi.nlm.nih.gov/pubmed/advanced) | Search "fibrohistiocytic tumour"[Title/Abstract] | [30](http://www.ncbi.nlm.nih.gov/pubmed/?cmd=HistorySearch&querykey=115) |
| [#114](http://www.ncbi.nlm.nih.gov/pubmed/advanced) | [Add](http://www.ncbi.nlm.nih.gov/pubmed/advanced) | Search "fibrohistiocytic tumor"[Title/Abstract] | [116](http://www.ncbi.nlm.nih.gov/pubmed/?cmd=HistorySearch&querykey=114) |
| [#112](http://www.ncbi.nlm.nih.gov/pubmed/advanced) | [Add](http://www.ncbi.nlm.nih.gov/pubmed/advanced) | Search "sarcoma ovarian"[Title/Abstract] | [1](http://www.ncbi.nlm.nih.gov/pubmed/?cmd=HistorySearch&querykey=112) |
| [#111](http://www.ncbi.nlm.nih.gov/pubmed/advanced) | [Add](http://www.ncbi.nlm.nih.gov/pubmed/advanced) | Search ovarian sarcoma*[Title/Abstract] | [97](http://www.ncbi.nlm.nih.gov/pubmed/?cmd=HistorySearch&querykey=111) |
| [#108](http://www.ncbi.nlm.nih.gov/pubmed/advanced) | [Add](http://www.ncbi.nlm.nih.gov/pubmed/advanced) | Search uterine sarcoma*[Title/Abstract] | [1047](http://www.ncbi.nlm.nih.gov/pubmed/?cmd=HistorySearch&querykey=108) |
| [#106](http://www.ncbi.nlm.nih.gov/pubmed/advanced) | [Add](http://www.ncbi.nlm.nih.gov/pubmed/advanced) | Search "perivascular epithelioid cell"[Title/Abstract] | [410](http://www.ncbi.nlm.nih.gov/pubmed/?cmd=HistorySearch&querykey=106) |
| [#105](http://www.ncbi.nlm.nih.gov/pubmed/advanced) | [Add](http://www.ncbi.nlm.nih.gov/pubmed/advanced) | Search "Perivascular Epithelioid Cell Neoplasms"[Mesh] | [2811](http://www.ncbi.nlm.nih.gov/pubmed/?cmd=HistorySearch&querykey=105) |
| [#103](http://www.ncbi.nlm.nih.gov/pubmed/advanced) | [Add](http://www.ncbi.nlm.nih.gov/pubmed/advanced) | Search PEComa[Title/Abstract] | [358](http://www.ncbi.nlm.nih.gov/pubmed/?cmd=HistorySearch&querykey=103) |
| [#102](http://www.ncbi.nlm.nih.gov/pubmed/advanced) | [Add](http://www.ncbi.nlm.nih.gov/pubmed/advanced) | Search "tumor rhabdoid" | [2](http://www.ncbi.nlm.nih.gov/pubmed/?cmd=HistorySearch&querykey=102) |
| [#101](http://www.ncbi.nlm.nih.gov/pubmed/advanced) | [Add](http://www.ncbi.nlm.nih.gov/pubmed/advanced) | Search "Rhabdoid Tumor"[Title/Abstract] | [1055](http://www.ncbi.nlm.nih.gov/pubmed/?cmd=HistorySearch&querykey=101) |
| [#100](http://www.ncbi.nlm.nih.gov/pubmed/advanced) | [Add](http://www.ncbi.nlm.nih.gov/pubmed/advanced) | Search "Rhabdoid Tumor"[Mesh] | [1099](http://www.ncbi.nlm.nih.gov/pubmed/?cmd=HistorySearch&querykey=100) |
| [#96](http://www.ncbi.nlm.nih.gov/pubmed/advanced) | [Add](http://www.ncbi.nlm.nih.gov/pubmed/advanced) | Search "Desmoplastic small round"[Title/Abstract] | [535](http://www.ncbi.nlm.nih.gov/pubmed/?cmd=HistorySearch&querykey=96) |
| [#93](http://www.ncbi.nlm.nih.gov/pubmed/advanced) | [Add](http://www.ncbi.nlm.nih.gov/pubmed/advanced) | Search "Desmoplastic Small Round Cell Tumor"[Mesh] | [92](http://www.ncbi.nlm.nih.gov/pubmed/?cmd=HistorySearch&querykey=93) |
| [#90](http://www.ncbi.nlm.nih.gov/pubmed/advanced) | [Add](http://www.ncbi.nlm.nih.gov/pubmed/advanced) | Search "clear cell sarcomas"[Title/Abstract] | [100](http://www.ncbi.nlm.nih.gov/pubmed/?cmd=HistorySearch&querykey=90) |
| [#89](http://www.ncbi.nlm.nih.gov/pubmed/advanced) | [Add](http://www.ncbi.nlm.nih.gov/pubmed/advanced) | Search "clear cell sarcoma"[Title/Abstract] | [818](http://www.ncbi.nlm.nih.gov/pubmed/?cmd=HistorySearch&querykey=89) |
| [#88](http://www.ncbi.nlm.nih.gov/pubmed/advanced) | [Add](http://www.ncbi.nlm.nih.gov/pubmed/advanced) | Search "Sarcoma, Clear Cell"[Mesh] | [467](http://www.ncbi.nlm.nih.gov/pubmed/?cmd=HistorySearch&querykey=88) |
| [#85](http://www.ncbi.nlm.nih.gov/pubmed/advanced) | [Add](http://www.ncbi.nlm.nih.gov/pubmed/advanced) | Search ASPS[Title/Abstract] | [704](http://www.ncbi.nlm.nih.gov/pubmed/?cmd=HistorySearch&querykey=85) |
| [#84](http://www.ncbi.nlm.nih.gov/pubmed/advanced) | [Add](http://www.ncbi.nlm.nih.gov/pubmed/advanced) | Search synovioma*[Title/Abstract] | [343](http://www.ncbi.nlm.nih.gov/pubmed/?cmd=HistorySearch&querykey=84) |
| [#82](http://www.ncbi.nlm.nih.gov/pubmed/advanced) | [Add](http://www.ncbi.nlm.nih.gov/pubmed/advanced) | Search "sarcoma synovial"[Title/Abstract] | [3](http://www.ncbi.nlm.nih.gov/pubmed/?cmd=HistorySearch&querykey=82) |
| [#81](http://www.ncbi.nlm.nih.gov/pubmed/advanced) | [Add](http://www.ncbi.nlm.nih.gov/pubmed/advanced) | Search synovial sarcoma*[Title/Abstract] | [2916](http://www.ncbi.nlm.nih.gov/pubmed/?cmd=HistorySearch&querykey=81) |
| [#78](http://www.ncbi.nlm.nih.gov/pubmed/advanced) | [Add](http://www.ncbi.nlm.nih.gov/pubmed/advanced) | Search "Sarcoma, Synovial"[Mesh] | [2799](http://www.ncbi.nlm.nih.gov/pubmed/?cmd=HistorySearch&querykey=78) |
| [#76](http://www.ncbi.nlm.nih.gov/pubmed/advanced) | [Add](http://www.ncbi.nlm.nih.gov/pubmed/advanced) | Search mesenchymoma*[Title/Abstract] | [839](http://www.ncbi.nlm.nih.gov/pubmed/?cmd=HistorySearch&querykey=76) |
| [#75](http://www.ncbi.nlm.nih.gov/pubmed/advanced) | [Add](http://www.ncbi.nlm.nih.gov/pubmed/advanced) | Search "Mesenchymoma"[Mesh] | [1900](http://www.ncbi.nlm.nih.gov/pubmed/?cmd=HistorySearch&querykey=75) |
| [#72](http://www.ncbi.nlm.nih.gov/pubmed/advanced) | [Add](http://www.ncbi.nlm.nih.gov/pubmed/advanced) | Search MPNST[Title/Abstract] | [852](http://www.ncbi.nlm.nih.gov/pubmed/?cmd=HistorySearch&querykey=72) |
| [#71](http://www.ncbi.nlm.nih.gov/pubmed/advanced) | [Add](http://www.ncbi.nlm.nih.gov/pubmed/advanced) | Search "malignant granular cell tumor"[Title/Abstract] | [100](http://www.ncbi.nlm.nih.gov/pubmed/?cmd=HistorySearch&querykey=71) |
| [#68](http://www.ncbi.nlm.nih.gov/pubmed/advanced) | [Add](http://www.ncbi.nlm.nih.gov/pubmed/advanced) | Search ectomesenchymoma*[Title/Abstract] | [70](http://www.ncbi.nlm.nih.gov/pubmed/?cmd=HistorySearch&querykey=68) |
| [#66](http://www.ncbi.nlm.nih.gov/pubmed/advanced) | [Add](http://www.ncbi.nlm.nih.gov/pubmed/advanced) | Search GIST[Title/Abstract] | [5011](http://www.ncbi.nlm.nih.gov/pubmed/?cmd=HistorySearch&querykey=66) |
| [#65](http://www.ncbi.nlm.nih.gov/pubmed/advanced) | [Add](http://www.ncbi.nlm.nih.gov/pubmed/advanced) | Search "gastrointestinal stromal tumors"[Title/Abstract] | [3776](http://www.ncbi.nlm.nih.gov/pubmed/?cmd=HistorySearch&querykey=65) |
| [#64](http://www.ncbi.nlm.nih.gov/pubmed/advanced) | [Add](http://www.ncbi.nlm.nih.gov/pubmed/advanced) | Search "gastrointestinal stromal tumor"[Title/Abstract] | [2960](http://www.ncbi.nlm.nih.gov/pubmed/?cmd=HistorySearch&querykey=64) |
| [#63](http://www.ncbi.nlm.nih.gov/pubmed/advanced) | [Add](http://www.ncbi.nlm.nih.gov/pubmed/advanced) | Search "Gastrointestinal Stromal Tumors"[Mesh] | [4675](http://www.ncbi.nlm.nih.gov/pubmed/?cmd=HistorySearch&querykey=63) |
| [#60](http://www.ncbi.nlm.nih.gov/pubmed/advanced) | [Add](http://www.ncbi.nlm.nih.gov/pubmed/advanced) | Search lymphangiosarcoma*[Title/Abstract] | [295](http://www.ncbi.nlm.nih.gov/pubmed/?cmd=HistorySearch&querykey=60) |
| [#59](http://www.ncbi.nlm.nih.gov/pubmed/advanced) | [Add](http://www.ncbi.nlm.nih.gov/pubmed/advanced) | Search "Lymphangiosarcoma"[Mesh] | [392](http://www.ncbi.nlm.nih.gov/pubmed/?cmd=HistorySearch&querykey=59) |
| [#56](http://www.ncbi.nlm.nih.gov/pubmed/advanced) | [Add](http://www.ncbi.nlm.nih.gov/pubmed/advanced) | Search angiosarcoma*[Title/Abstract] | [5190](http://www.ncbi.nlm.nih.gov/pubmed/?cmd=HistorySearch&querykey=56) |
| [#53](http://www.ncbi.nlm.nih.gov/pubmed/advanced) | [Add](http://www.ncbi.nlm.nih.gov/pubmed/advanced) | Search haemangiosarcoma*[Title/Abstract] | [205](http://www.ncbi.nlm.nih.gov/pubmed/?cmd=HistorySearch&querykey=53) |
| [#52](http://www.ncbi.nlm.nih.gov/pubmed/advanced) | [Add](http://www.ncbi.nlm.nih.gov/pubmed/advanced) | Search hemangiosarcoma*[Title/Abstract] | [914](http://www.ncbi.nlm.nih.gov/pubmed/?cmd=HistorySearch&querykey=52) |
| [#51](http://www.ncbi.nlm.nih.gov/pubmed/advanced) | [Add](http://www.ncbi.nlm.nih.gov/pubmed/advanced) | Search "Hemangiosarcoma"[Mesh] | [6237](http://www.ncbi.nlm.nih.gov/pubmed/?cmd=HistorySearch&querykey=51) |
| [#48](http://www.ncbi.nlm.nih.gov/pubmed/advanced) | [Add](http://www.ncbi.nlm.nih.gov/pubmed/advanced) | Search hemangioendothelioma*[Title/Abstract] | [2493](http://www.ncbi.nlm.nih.gov/pubmed/?cmd=HistorySearch&querykey=48) |
| [#47](http://www.ncbi.nlm.nih.gov/pubmed/advanced) | [Add](http://www.ncbi.nlm.nih.gov/pubmed/advanced) | Search haemangioendothelioma*[Title/Abstract] | [367](http://www.ncbi.nlm.nih.gov/pubmed/?cmd=HistorySearch&querykey=47) |
| [#46](http://www.ncbi.nlm.nih.gov/pubmed/advanced) | [Add](http://www.ncbi.nlm.nih.gov/pubmed/advanced) | Search "Hemangioendothelioma"[Mesh] | [3308](http://www.ncbi.nlm.nih.gov/pubmed/?cmd=HistorySearch&querykey=46) |
| [#41](http://www.ncbi.nlm.nih.gov/pubmed/advanced) | [Add](http://www.ncbi.nlm.nih.gov/pubmed/advanced) | Search rhabdomyosarcoma*[Title/Abstract] | [10291](http://www.ncbi.nlm.nih.gov/pubmed/?cmd=HistorySearch&querykey=41) |
| [#40](http://www.ncbi.nlm.nih.gov/pubmed/advanced) | [Add](http://www.ncbi.nlm.nih.gov/pubmed/advanced) | Search "Myosarcoma"[Mesh] | [9866](http://www.ncbi.nlm.nih.gov/pubmed/?cmd=HistorySearch&querykey=40) |
| [#34](http://www.ncbi.nlm.nih.gov/pubmed/advanced) | [Add](http://www.ncbi.nlm.nih.gov/pubmed/advanced) | Search glomangiosarcoma*[Title/Abstract] | [32](http://www.ncbi.nlm.nih.gov/pubmed/?cmd=HistorySearch&querykey=34) |
| [#33](http://www.ncbi.nlm.nih.gov/pubmed/advanced) | [Add](http://www.ncbi.nlm.nih.gov/pubmed/advanced) | Search leiomyosarcoma*[Title/Abstract] | [8823](http://www.ncbi.nlm.nih.gov/pubmed/?cmd=HistorySearch&querykey=33) |
| [#32](http://www.ncbi.nlm.nih.gov/pubmed/advanced) | [Add](http://www.ncbi.nlm.nih.gov/pubmed/advanced) | Search "Leiomyosarcoma"[Mesh] | [7920](http://www.ncbi.nlm.nih.gov/pubmed/?cmd=HistorySearch&querykey=32) |
| [#30](http://www.ncbi.nlm.nih.gov/pubmed) | [Add](http://www.ncbi.nlm.nih.gov/pubmed) | Search MFH[Title/Abstract] | [1336](http://www.ncbi.nlm.nih.gov/pubmed/?cmd=HistorySearch&querykey=30) |
| [#29](http://www.ncbi.nlm.nih.gov/pubmed) | [Add](http://www.ncbi.nlm.nih.gov/pubmed) | Search "Histiocytoma, Malignant Fibrous"[Mesh] | [701](http://www.ncbi.nlm.nih.gov/pubmed/?cmd=HistorySearch&querykey=29) |
| [#28](http://www.ncbi.nlm.nih.gov/pubmed) | [Add](http://www.ncbi.nlm.nih.gov/pubmed) | Search hystiocytoma*[Title/Abstract] | [9](http://www.ncbi.nlm.nih.gov/pubmed/?cmd=HistorySearch&querykey=28) |
| [#27](http://www.ncbi.nlm.nih.gov/pubmed) | [Add](http://www.ncbi.nlm.nih.gov/pubmed) | Search histiocytoma*[Title/Abstract] | [5306](http://www.ncbi.nlm.nih.gov/pubmed/?cmd=HistorySearch&querykey=27) |
| [#22](http://www.ncbi.nlm.nih.gov/pubmed) | [Add](http://www.ncbi.nlm.nih.gov/pubmed) | Search "Hemangiopericytoma"[Mesh] | [2663](http://www.ncbi.nlm.nih.gov/pubmed/?cmd=HistorySearch&querykey=22) |
| [#19](http://www.ncbi.nlm.nih.gov/pubmed) | [Add](http://www.ncbi.nlm.nih.gov/pubmed) | Search hemangiopericytoma*[Title/Abstract] | [2556](http://www.ncbi.nlm.nih.gov/pubmed/?cmd=HistorySearch&querykey=19) |
| [#18](http://www.ncbi.nlm.nih.gov/pubmed) | [Add](http://www.ncbi.nlm.nih.gov/pubmed) | Search haemangiopericytoma*[Title/Abstract] | [491](http://www.ncbi.nlm.nih.gov/pubmed/?cmd=HistorySearch&querykey=18) |
| [#17](http://www.ncbi.nlm.nih.gov/pubmed) | [Add](http://www.ncbi.nlm.nih.gov/pubmed) | Search myxosarcoma*[Title/Abstract] | [239](http://www.ncbi.nlm.nih.gov/pubmed/?cmd=HistorySearch&querykey=17) |
| [#16](http://www.ncbi.nlm.nih.gov/pubmed) | [Add](http://www.ncbi.nlm.nih.gov/pubmed) | Search "Myxosarcoma"[Mesh] | [380](http://www.ncbi.nlm.nih.gov/pubmed/?cmd=HistorySearch&querykey=16) |
| [#13](http://www.ncbi.nlm.nih.gov/pubmed) | [Add](http://www.ncbi.nlm.nih.gov/pubmed) | Search myxofibrosarcoma*[Title/Abstract] | [348](http://www.ncbi.nlm.nih.gov/pubmed/?cmd=HistorySearch&querykey=13) |
| [#12](http://www.ncbi.nlm.nih.gov/pubmed) | [Add](http://www.ncbi.nlm.nih.gov/pubmed) | Search dermatofibrosarcoma*[Title/Abstract] | [1584](http://www.ncbi.nlm.nih.gov/pubmed/?cmd=HistorySearch&querykey=12) |
| [#11](http://www.ncbi.nlm.nih.gov/pubmed) | [Add](http://www.ncbi.nlm.nih.gov/pubmed) | Search fibrosarcoma*[Title/Abstract] | [15965](http://www.ncbi.nlm.nih.gov/pubmed/?cmd=HistorySearch&querykey=11) |
| [#10](http://www.ncbi.nlm.nih.gov/pubmed) | [Add](http://www.ncbi.nlm.nih.gov/pubmed) | Search "Fibrosarcoma"[Mesh] | [12585](http://www.ncbi.nlm.nih.gov/pubmed/?cmd=HistorySearch&querykey=10) |
| [#6](http://www.ncbi.nlm.nih.gov/pubmed) | [Add](http://www.ncbi.nlm.nih.gov/pubmed) | Search "Liposarcoma"[Mesh] | [4128](http://www.ncbi.nlm.nih.gov/pubmed/?cmd=HistorySearch&querykey=6) |
| [#3](http://www.ncbi.nlm.nih.gov/pubmed) | [Add](http://www.ncbi.nlm.nih.gov/pubmed) | Search liposarcoma*[Title/Abstract] | [6164](http://www.ncbi.nlm.nih.gov/pubmed/?cmd=HistorySearch&querykey=3) |
| [#2](http://www.ncbi.nlm.nih.gov/pubmed) | [Add](http://www.ncbi.nlm.nih.gov/pubmed) | Search sarcoma[mh:noexp] | [27579](http://www.ncbi.nlm.nih.gov/pubmed/?cmd=HistorySearch&querykey=2) |
| [#1](http://www.ncbi.nlm.nih.gov/pubmed) | [Add](http://www.ncbi.nlm.nih.gov/pubmed) | Search sarcoma*[Title/Abstract] |  |

Search strategy:

Database: Embase <1980 to 2016 Week 14>

Search Strategy:

--------------------------------------------------------------------------------

1 sarcoma*.ti,ab. (89317)

2 *sarcoma/ (16778)

3 liposarcoma*.ti,ab. (6226)

4 exp liposarcoma/ (6521)

5 exp fibrosarcoma/ (12565)

6 fibrosarcoma*.ti,ab. (10584)

7 dermatofibrosarcoma*.ti,ab. (1987)

8 myxofibrosarcoma*.ti,ab. (474)

9 exp myxosarcoma/ (1663)

10 myxosarcoma*.ti,ab. (167)

11 haemangiopericytoma*.ti,ab. (533)

12 hemangiopericytoma*.ti,ab. (2799)

13 exp hemangiopericytoma/ (3770)

14 histiocytoma*.ti,ab. (5923)

15 hystiocytoma*.ti,ab. (19)

16 exp malignant fibrous histiocytoma/ (3812)

17 MFH.ti,ab. (1571)

18 exp leiomyosarcoma/ (11819)

19 leiomyosarcoma.ti,ab. (9031)

20 glomangiosarcoma*.ti,ab. (36)

21 exp myosarcoma/ (349)

22 rhabdomyosarcoma*.ti,ab. (11879)

23 exp hemangioendothelioma/ (3805)

24 haemangioendothelioma*.ti,ab. (434)

25 hemangioendothelioma*.ti,ab. (2699)

26 exp angiosarcoma/ (8240)

27 hemangiosarcoma*.ti,ab. (814)

28 haemangiosarcoma*.ti,ab. (199)

29 angiosarcoma*.ti,ab. (5908)

30 exp lymphangiosarcoma/ (424)

31 lymphangiosarcoma*.ti,ab. (281)

32 exp gastrointestinal stromal tumor/ (11877)

33 "gastrointestinal stromal tumor".ti,ab. (3864)

34 "gastrointestinal stromal tumors".ti,ab. (5048)

35 gist.ti,ab. (7672)

36 ectomensenchymoma.ti,ab. (0)

37 "malignant granular cell tumor".ti,ab. (112)

38 mpnst.ti,ab. (1146)

39 exp mesenchymoma/ (2511)

40 mesenchymoma*.ti,ab. (532)

41 exp synovial sarcoma/ (4047)

42 synovial sarcoma*.ti,ab. (3606)

43 "sarcoma synovial".ti,ab. (57)

44 synovioma*.ti,ab. (184)

45 ASPS.ti,ab. (865)

46 exp clear cell sarcoma/ (1174)

47 "clear cell sarcoma".ti,ab. (967)

48 "clear cell sarcomas".ti,ab. (120)

49 exp desmoplastic small round cell tumor/ (767)

50 "desmoplastic small round".ti,ab. (675)

51 exp rhabdoid tumor/ (2331)

52 "rhabdoid tumor".ti,ab. (1434)

53 "tumor rhabdoid".ti,ab. (15)

54 pecoma.ti,ab. (452)

55 "perivascular epithelioid cell".ti,ab. (452)

56 exp perivascular epithelioid cell tumor/ (491)

57 exp uterus sarcoma/ (2170)

58 uterine sarcoma*.ti,ab. (1418)

59 ovarian sarcoma*.ti,ab. (106)

60 "sarcoma ovarian".ti,ab. (15)

61 "fibrohistiocytic tumor".ti,ab. (131)

62 myofibroblastic sarcoma*.ti,ab. (144)

63 epithelioid sarcoma*.ti,ab. (898)

64 exp endometrium sarcoma/ (1379)

65 endometrial sarcoma*.ti,ab. (192)

66 "endometrial stromal sarcoma".ti,ab. (1041)

67 1 or 2 or 3 or 4 or 5 or 6 or 7 or 8 or 9 or 10 or 11 or 12 or 13 or 14 or 15 or 16 or 17 or 18 or 19 or 20 or 21 or 22 or 23 or 24 or 25 or 26 or 27 or 28 or 29 or 30 or 31 or 32 or 33 or 34 or 35 or 36 or 37 or 38 or 39 or 40 or 41 or 42 or 43 or 44 or 45 or 46 or 47 or 48 or 49 or 50 or 51 or 52 or 53 or 54 or 55 or 56 or 57 or 58 or 59 or 60 or 61 or 62 or 63 or 64 or 65 or 66 (167542)

68 kaposi.ti. (1657)

69 ewing.ti. (2007)

70 68 or 69 (3664)

71 limit 67 to ("systematic review" and yr="1990 - 2016") (502)

74 67 not 70 (164347)

75 limit 74 to "systematic review" (494)

Database: Cochrane Library

Date Run: 05/04/16 11:04:53.538

ID Search Hits

#1 sarcoma* 1461

#2 MeSH descriptor: [Sarcoma] this term only 305

#3 lipomatous next tumour 0

#4 liposarcoma* 52

#5 MeSH descriptor: [Liposarcoma] explode all trees 6

#6 liposarcoma next myxoid 2

#7 dermatofibrosarcoma* 12

#8 fibrosarcoma* 34

#9 MeSH descriptor: [Fibrosarcoma] explode all trees 8

#10 myofibroblastic next sarcoma* 0

#11 myxofibrosarcoma* 3

#12 myxosarcoma* 9

#13 MeSH descriptor: [Myxosarcoma] explode all trees 0

#14 fibromyxoid next sarcoma* 1

#15 epithelioid next fibrosarcoma* 1

#16 haemangiopericytoma* 1

#17 hemagiopericytoma* 0

#18 MeSH descriptor: [Hemangiopericytoma] explode all trees 2

#19 hystiocytoma* 0

#20 histiocytoma* 27

#21 MeSH descriptor: [Histiocytoma, Malignant Fibrous] explode all trees 0

#22 histiocytoma near/3 malignant 17

#23 MFH 9

#24 leiomyosarcoma* 103

#25 MeSH descriptor: [Leiomyosarcoma] explode all trees 29

#26 glomagiosarcoma* 0

#27 glomus near/2 tumour* 12

#28 rhabdomyosarcoma* 183

#29 MeSH descriptor: [Myosarcoma] explode all trees 60

#30 kaposiform near/2 hemangioendothelioma* 0

#31 hemangioendothelioma* 7

#32 haemangioendothelioma* 0

#33 MeSH descriptor: [Hemangioendothelioma] explode all trees 2

#34 haemangiosarcoma* 2

#35 hemangiosarcoma* 8

#36 MeSH descriptor: [Hemangiosarcoma] explode all trees 2

#37 epithelioid next sarcoma* 3

#38 angiosarcoma* 22

#39 lymphangiosarcoma* 5

#40 MeSH descriptor: [Lymphangiosarcoma] explode all trees 1

#41 gastrointestinal next stromal next tumours 51

#42 gastrointestinal next stromal next tumors 151

#43 gist 247

#44 MeSH descriptor: [Gastrointestinal Stromal Tumors] explode all trees 112

#45 ectomesenchymoma* 1

#46 malignant next granular next cell 0

#47 MPNST 2

#48 mesenchymoma* 5

#49 MeSH descriptor: [Mesenchymoma] explode all trees 2

#50 MeSH descriptor: [Sarcoma, Synovial] explode all trees 0

#51 sarcoma next synovial 2

#52 synovial next sarcoma* 23

#53 synovioma* 0

#54 ASPS 14

#55 sarcoma near/2 clear cell 14

#56 MeSH descriptor: [Sarcoma, Clear Cell] this term only 3

#57 "desmoplastic small round cell tumor" 4

#58 desmoplastic next small round cell tumo* 5

#59 rhabdoid next tumo* 13

#60 MeSH descriptor: [Rhabdoid Tumor] explode all trees 1

#61 PEComa 1

#62 perivascular next epithelioid next cell 1

#63 MeSH descriptor: [Perivascular Epithelioid Cell Neoplasms] explode all trees 13

#64 uterine near/2 sarcoma* 35

#65 ovarian near/2 sarcoma* 5

#66 fibrohistiocytic next tumo* 0

#67 #1 or #2 or #3 or #4 or #5 or #6 or #7 or #8 or #9 or #10 or #11 or #12 or #14 or #15 or #16 or #17 or #19 or #20 or #21 or #22 or #23 or #24 or #25 or #26 or #27 or #28 or #29 or #30 or #31 or #32 or #34 or #35 or #37 or #38 or #39 or #41 or #42 or #43 or #45 or #46 or #47 or #48 or #49 or #50 or #51 or #52 or #53 or #54 or #55 or #56 or #57 or #58 or #59 or #60 or #61 or #62 or #63 or #64 or #65 1941

#76 Ewing*:ti 114

#77 kaposi:ti 120

#78 #76 or #77 234

#79 #67 not #78 Publication Year from 2000 to 2015 1153

All Results (1153)

Cochrane Reviews (190)

Other Reviews (76)

Trials (772)

Methods Studies (6)

Technology Assessments (68)

Economic Evaluations (35)

Cochrane Groups (6)

Search strategy Epistemonikos:

title:(sarcoma* OR liposarcoma* OR dermatofibrosarcoma* OR fibrosarcoma* OR myxofibrosarcoma* OR haemangiopericytoma OR histiocytoma* OR MFH OR leiomyosarcoma* OR glomangiosarcoma* OR rhabdomyosarcoma* OR hemangioendothelioma* OR haemangiosarcoma* OR hemangiosarcoma* OR angiosarcoma* lymphangiosarcoma* OR (gastrointestinal AND stromal AND (tumor OR tumour OR neoplas*)) OR GIST OR ectomesenchymoma* OR (malignant AND (“granular cell” OR “granular cells”)) OR MPNST OR mesenchymoma* OR ASPS OR (desmoplastic AND round) OR (rhabdoid AND (tumor OR tumour OR neoplas*)) OR PEComa OR (perivascular AND epithelioid AND (tumor OR tumour OR neoplas*)) OR (fibrohistiocytic AND (tumor OR tumour OR neoplas*)) OR (myofibroblastic AND sarcoma) OR (fibromyxoid AND sarcoma) OR (epithelioid AND fibrosarcoma)) OR abstract:(sarcoma* or liposarcoma* OR dermatofibrosarcoma* OR fibrosarcoma* OR myxofibrosarcoma* OR haemangiopericytoma OR histiocytoma* OR MFH OR leiomyosarcoma* OR glomangiosarcoma* OR rhabdomyosarcoma* OR hemangioendothelioma* OR haemangiosarcoma* OR hemangiosarcoma* OR angiosarcoma* OR lymphangiosarcoma* OR "gastrointestinal stromal tumor" OR (gastrointestinal AND stromal AND (tumor OR tumour OR neoplas*)) OR GIST OR ectomesenchymoma* OR (malignant AND (“granular cell” OR “granular cells”)) OR MPNST OR mesenchymoma* OR ASPS OR (desmoplastic AND round) OR (rhabdoid AND (tumor OR tumour OR neoplas*)) OR PEComa OR (perivascular AND epithelioid AND (tumor OR tumour OR neoplas*)) OR (myofibroblastic AND sarcoma) OR (fibromyxoid AND sarcoma) OR (epithelioid AND fibrosarcoma))
