# Supplementary material for: Evidence mapping based on systematic reviews of therapeutic interventions for gastrointestinal stromal tumors (GIST)
Source: BMC Med Res Methodol. 2017 Sep 7;17:135. doi: 10.1186/s12874-017-0402-9 (PMC5590134; doi:10.1186/s12874-017-0402-9)
Supplement: Supplementary file 2 — SRs excluded. (DOCX 18 kb) [file 12874_2017_402_MOESM2_ESM.docx]

**Additional file 2**

| Study | Reason for exclusion |
| --- | --- |
| Abernethy 2005 | Article withdrawn due to lack of update |
| Abhyankar 2013 | Conference paper, full text not available |
| Afonso 2010 | Poster, full text not available |
| Ahram 2006 | Comprehensive literature review not conducted. Narrative review |
| Akbulut 2014 | Therapeutic interventions were not assessed |
| Athale 2009 | Rhabdoid tumor is not considered as sarcoma. Narrative review |
| Avancès 2011 | Narrative review, only one database was used for literature search |
| Avancès 2013 | Recommendations, no systematic review |
| AWTTC 2014 | Results reported in other included study (NICE 2014) |
| Benesch 2009 | Literature search limited to one database |
| Bernstein 2015 | Literature search limited to one database. Narrative review |
| Blay 2009 | Narrative review |
| Blay 2011 | Narrative review |
| Budach 1996 | Opinion article |
| Chen 2010 | Therapeutic interventions were not assessed |
| Chen 2015 | Previous version other systematic review included (Chen 2014) |
| Cheng 2011 | Therapeutic interventions were not assessed |
| Chok 2014 | Literature search limited to one database |
| Conesa 2011 | Literature search limited to one database |
| Dafopoulos 2010 | Narrative review |
| Dahodwala 2013 | Literature search limited to one database |
| Dalia 2014 | Narrative review |
| Dangle 2016 | Literature search limited to one database. Narrative review |
| Debled 1997 | Conference paper. Full text not available |
| Delporte 2011 | Literature search limited to one database |
| Demetri 2010 | Focus on providing treatmentrecommendations |
| Deppe 2014 | Therapeutic interventions were not assessed |
| Desai 2015 | Conference paper. Full text not available |
| Dhir 2014 | Therapeutic interventions were not assessed |
| Dretzke 2010 | Technology assessment based on systematic review conducted by industry. Incomplete data were presented in the report. |
| Ducoulombier 2016 | Literature search limited to one database |
| Duval 2013 | Hemangioperycitoma is not classified as soft tissue sarcoma. |
| ElDibRegina 2012 | Only protocol is available. Systematic review not conducted |
| Fort 2016 | Systematic review focused on Kaposi tumour exclusively |
| Fra 2004 | Narrative review |
| Froehner 2014 | Therapeutic interventions were not assessed |
| Gadducci 2015 | Narrative review based on expert opinion |
| Gilg 2016 | Therapeutic interventions were not assessed |
| Haas 2015 | Literature search limited to one database. Narrative review |
| Healy 2010 | Literature search limited to one database |
| Hislop 2011 | This study reported the same data than Hislop 2012 |
| Hoekstra 2004 | Narrative review |
| Horazdovsky 2013 | Literature search limited to one database. Rhabdoid tumor is not considered as sarcoma |
| Howard 2015 | Langerhans cell sarcoma not is considered as true sarcoma |
| Iavazzo 2014 | Narrative review |
| Jafari 2013 | Literature search limited to one database |
| Jain 2009 | Literature search limited to one database |
| Jerraya 2009 | Literature search limited to one database |
| Karligkiotis 2013 | Case series review (6 cases). Literature search limited to one database |
| Koh 2013 | Literature search limited to one database |
| Kuderer 2013 | Systematic review focused on solid tumours in general, no just sarcoma |
| Lai 2012 | Narrative review |
| LeCesne 2009 | Literature search limited to one database |
| Madge 2010 | Literature search limited to one database |
| Mali 2013 | Study focused in any kind of tumour. |
| Mattox 2010 | Literature search limited to one database |
| McConnell 2012 | Literature search limited to one database |
| Mendenhall 2005 | Narrative review |
| Michot 2014 | Literature search limited to one database. Details about search and selection of studies were not reported |
| Miller 2012 | Recommendations, no systematic review |
| Musella 2015 | Therapeutic interventions were not assessed |
| Nagib 2005 | only available in abstract format |
| National Horizon Scanning Centre 2006 | Literature search limited to one database. The search was described as limited |
| Ngan 2013 | Literature search limited to one database |
| NHSC 2008 | The search was described as limited |
| NICE 2010 | This systematic review reported the same data of Hislop 2012 |
| NIHR 2012 | The search was described as limited |
| Ntourakis 2015 | Literature search limited to one database. Other types of tumours differents to sarcoma were included |
| Oliveira 2015 | Narrative review of eight cases |
| Ossendorf 2008 | Therapeutic interventions were not assessed |
| Padhi 2013 | Therapeutic interventions were not assessed |
| Pallure 2013 | Literature search limited to one database |
| PichonRiviere 2006 | Therapeutic interventions were not assessed |
| Reid 2013 | Narrative review |
| Rusthoven 2014 | Therapeutic interventions were not assessed. Literature search limited to one database |
| Rutkowski 2010 | Literature search limited to one database |
| Rutkowski 2013 | Narrative review. Methodology details not provided |
| Salim 2012 | Conference paper. Full text not available |
| Samelis 2007 | Narrative review |
| Sampath 2011 | Narrative review |
| Sarcoma Metanalisis collaboration 2000 | An update of this review was included (Pervaiz 2008) |
| Sbitti 2011 | Literature search limited to one database |
| Schöffski 2014 | Narrative review |
| Schrey 2014 | Rhabdoid tumor is not considered as a true sarcoma. |
| Scoggins 2005 | Narrative review |
| Shah 2015 | Conference paper. Full text not available |
| Sheth 2012 | Therapeutic interventions were not assessed. Literature search limited to one database |
| Sicklick 2013 | Narrative review |
| Strander 2003 | Literature search limited to one database |
| Thornton 2009 | Therapeutic comparison were not found |
| Tierney 1995 | An update of this review was included (Pervaiz 2008) |
| Tropé 2012 | Narrative review. No therapeutic interventions were assessed |
| Tuan 2014 | Literature search limited to one database |
| Vennarini 2014 | Poster. Full text not available |
| Weigel B | Literature search limited to one database |
| Wushou 2015 | Hemangioperycitoma is not classified as soft tissue sarcoma. |
| Xu 2013 | Therapeutic interventions were not assessed |
| Xu 2015 | Includes soft and bone sarcomas. Chondrosarcoma is not soft tissue sarcoma |
| Ye 2009 | Narrative review |
| Yilmaz 2013 | Narrative review |
| Yin 2010 | Focus on leiomyosarcoma and leiomyoma in HIV positive patients |
| Zhou 2014 | Conference paper. Full text not available. |
